# Synergy effect of science and technology policies on innovation: Evidence from China

**DOI:** 10.1371/journal.pone.0240515

**Published:** 2020-10-13

**Authors:** Shoulin Pang, Shiting Dou, Huan Li

**Affiliations:** Business School, Central University of Finance and Economics, Beijing, China; China University of Mining and Technology, CHINA

## Abstract

This paper explores the synergy effect of the government subsidies, tax incentives, and government procurement on innovation based on synergy theory, and further analyzes its path and mechanism in the process of innovation. We find that government subsidies, tax incentives, and government procurement exert positive synergy effect on innovation. Furthermore, in the process of innovation, government subsidies are shown to play strongest roles in the stages of innovation input and technological development, while government procurement is the most crucial in the transformation stage of technological innovation, and tax incentives play balanced roles. We also find that innovation resource input, innovation technology spillover, and innovation cooperation all play partial mediating roles in the synergy effect of science and technology policies on innovation. This paper applies the synergy theory to the field of innovation policies, which enriches and expands relevant researches, and provides micro-evidence for in-depth understanding of the effect of science and technology policies on innovation.

## Introduction

As the “new engine” of economic development, innovation plays an increasingly important role in the economic development of countries around the world [1]. In particular, as the largest emerging economy, China's innovation developed rapidly and achieved remarkable results, and the determining factors of such rapid development have become a popular research topic, especially the science and technology policies. The Chinese government has established a relatively comprehensive system of policies to promote the marketization of innovation, such as subsidies, tax incentives, and government procurement-all of which are meant to stimulate innovation synergisticly. However, policies may be substituted, which makes the implementation effects of policies deviating from the goals [2]. Therefore, how to deal with the synergy effect of policies in the process of innovation constitutes one of the challenges for policy making [3].

Policy synergy is the result of the conflict or competition occurring among different policies, and is an effective way to resolve policy conflict [4]. Policy synergy is beneficial to improving the efficiency of policies and realizing pareto optimal [5]. Moreover, excessive emphasis on any single policy can be detrimental to the promotion of innovation [6], only when a variety of science and technology policies are properly nested and coordinated can policy goals be achieved. However, there is insufficient research on the synergy effect of science and technology policies. Then, does the synergy effect of science and technology policies on innovation in china exist? In the process of innovation, what is the path and mechanism of the synergy effect? What is the meaning of the synergy effect for policy adjustment? The exploration of these problems will provide important insights for policy adjustment and the development of innovation in China and even the whole world.

Based on synergy theory, we explore the synergy effect of science and technology policies on innovation, and further analyze its path and mechanism. Among policies, government subsidies, tax incentives and government procurement are enterprise-level policies, and considering the scale of policies and data availability, we focuses on the three policies. The contributions of this paper are as the following: Firstly, on the basis of existing research, we analyze the theoretical mechanisms of the synergy effect of science and technology policies based on synergy theory, which enriches related researches, and expands the application of synergy theory. Secondly, we divide innovation into three stages (innovation input, technology development, and achievement transformation) according to the innovation cycle in order to analyze the path of the synergy effect, and find that different policies have different effects on different stages of innovation, which has a certain guiding significance for policy formulation and adjustment. Thirdly, with attention to the internal and external enterprises behaviors in the process of innovation, we analyze the mechanisms of the synergy effect of science and technology policies on innovation, and we find that innovation resource input, innovation technology spillover, and innovation cooperation all play partial mediating roles, which provide new insights relevant to related research.

## Literature review

### Single form science and technology policies and innovation

Government subsidies. The imperfections of the capital market, the high risks and uncertainties of innovation lead enterprises to face serious financing constraints and hinder the process of innovation [7]. Government subsidies can help alleviate the financing constraints, diversify the risks of innovation activities, directly reduce the marginal cost of technological innovation, and increase innovation inputs and outputs [8–11]. However, information asymmetry between enterprises and governments is likely to cause *ex ante* adverse selection and *ex post* moral hazard when applying for subsidies [12]. Moreover, if the government lacks effective regulatory system and screening mechanism, enterprises may intentionally commit subsidy fraud by sending false signals to the government [13], which breeds “rent-seeking” behavior [14]. In addition, governing government subsidy programs only allow enterprises to apply for projects within said programs, compelling them to forego possibly more valuable investment opportunities. As a result, government subsidies enable enterprises to abort original projects, and crowd out innovation input [15]. However, some scholars believe that government subsidies are “ineffective” for innovation [16, 17].

Tax incentives (such as pre-tax deductions for R&D expenditures, accelerated depreciation, and income tax concessions) can directly reduce innovation costs, encourage enterprises to increase R&D expenditures [18, 19], and improve the innovation performance [20, 21]. However, Bloom et al. [22] showed that tax incentives reduced enterprises’ R&D costs by 10 percent; however, enterprises’ R&D investment only increased by about 1 percent in the short term, and no more than 10 percent in the long term. Scholars attempted to apply "crowding out effects" to explain the inhibition effects of tax incentives on innovation, they thought that the tax incentives for low-tech products crowded out the investment of high-tech products [23], and the insufficient implementation of tax incentives [20], the subjective tax avoidance, manipulation of research and development, the improper risk sharing all affect the effects of tax incentives [24].

As a demand-side policy, government procurement can help to solve market failure and system failure in innovation activities by identifying, expressing and successfully integrating fragmented individual needs [25]. In particular, government procurement can create demand for innovative services or products [26], and establish pilot markets [27, 28], which will encourage enterprises to increase R&D investment in order to capture this market, and gain a full competitive advantage by introducing advanced science and technology, and improve the ability of independent innovation [29, 30]. Meanwhile, government procurement can stimulate innovation by attracting the attention of investors [31] and easing corporate financing constraints [32]. Therefore, it has become an important policy to stimulate innovation [33, 34].

### Synergy effect of science and technology policies on innovation

With the development of policy research, many scholars began to question the research validity of single policy, and proposed that enterprises can benefit from multiple policies at the same time, if we do not control other policies, and only analyze a single policy will lead to potential bias [35].

Some scholars have studied the effects of multiple policies on innovation. Hægeland [36] found that government subsidies and tax incentives were complementary at the enterprise level and antagonistic at the innovation level. Chen [37] found that Chinese fiscal and tax incentives had complementary effects on R&D investment, and subsidies were more effective than tax incentives. Aschhoff and Sofka [33] revealed that public procurement, subsidies, knowledge spillovers, regulations, and innovation public procurement had significant incentive effects on innovation. Guerzoni and Raiteri [38] studied the impact of single policies as well as the interaction between two and three policies on innovation. They found that the incentive effect of government procurement was stronger than that of government subsidies and tax incentives, they also concluded that multiple policies were more effective than single policy. Montmartin [39] found that French government subsidies could produce significant incentive effects, and tax credits, local subsidies, and European subsidies tended to produce beggar-thy-neighbour effects. Fernández Sastre [40] argued that public procurement did not motivate enterprises to invest in R&D activities, while participation in innovation support programs had a significant incentive effect. Moreover, the combination of the two policies showed no significant influence on R&D decisions.

The above mentioned research considered multiple science and technology policies, but they only studied the impact of two or three policies on enterprise technology innovation alone without conducting in-depth analysis of the interaction among policies. However, some studies considered the interaction among policies, Zhu [41] found that government grants and tax incentives complemented and promoted each other, and the incentive effects were dominated mainly by government tax incentives. Kalcheva et al. [42] studied the effects of supply-side environment and demand-side policies on innovation by applying a triple-difference method, they found that the better the supply-side environment is, the stronger the impacts of demand-side policies on innovation are. Han and Ma [2] separated tax incentives into R&D expenses plus deductions, tax rate incentives, and accelerated depreciation of fixed assets, and found that different tax incentives had different incentive effects on R&D investment, and the incentive effects offset each other when multiple tax incentives were implemented simultaneously.

Indeed, some progress has been achieved in the research, however, there are still some certain limitations: Firstly, the procedural, staged and complex nature of innovation means that the forms and instruments of government support are diverse. Therefore, analysis of single policy will ignore the role of other policies and lead to biased and partial results. Secondly, although some scholars are aware of the interactions among policies, most scholars analyzed policies independently while ignoring the deep interactions among policies, and few studies considered the role of government procurement in China. Government subsidies, tax incentives, and government procurement policies promote and complement each other, and stimulate innovation synergisticly. However, there is little research on this topic. Thirdly, most research neglect the mechanism of policies to encourage innovation, more concerning, research on mechanism from the perspective of internal innovation resource input, technology spillover and external innovation cooperation does not exist.

## Theory

### Theoretical foundation

Synergy theory is an important component of systematic theory. Haken first attempted a systematic explanation of synergy theory in 1976, and pointed out that when external energy or the aggregation state of matter reached a certain critical value, any complex system would transform from disorder to order, resulting in synergy effect [43]. Synergy theory mainly studies the relationship among various elements of a system, elements and systems, and systems and environments [44, 45]. Synergy theory is widely used in economy and management. With the increasing complexity of the policy operating environment and the need to deal with multiple issues jointly, such as international competition, trade friction, and economic crisis [46], scholars are beginning to identify the importance of synergy theory for policy analysis [47].

Policy synergy refers to the coordination of multiple policies to achieve different policy objectives so as to improve the efficiency of policies and achieve Pareto optimality [5, 48]. When implementing policies, they are often unstable due to changes in the environment. In this situation, a synergistic combination of policies can achieve better results [3]. Therefore, the government should utilize policy synergy to maintain policy stability [49]. In the process of innovation, science and technology policies promote and complement each other, and the synergy effect of government subsidies, tax incentives and government procurement are mainly reflected in the following:

①Promotion mechanism. Government subsidies provide enterprises with innovation resources and alleviate financing constrains [11], tax incentives reduce the tax burden of innovation activities and increase expected benefits [21], government procurement can stimulate considerable market demand and drives innovation with demand [33]. The three policies give enterprises confidence, and work together to encourage enterprises’ innovation, expand the implementation effects of scientific and technological policies and realize synergy effect [41].

②Supplementary mechanism. Government subsidies can compensate for market failures in the process of research and development and achieve the growth effect of innovation investment. However, subsidies may cause unfair competition in the market, resulting in unchecked competition and uncontrolled “rent-seeking” [12]. Tax incentives are universal, do not interfere with market mechanisms, and can maximize the effectiveness of the market in the allocation of resources [41]. However, these two policies only serve guiding roles from the supply-side of enterprises, and cannot stimulate enterprises to participate in innovation from demand side. Government procurement can complement the demand side incentives of policies, compensating for the limitations of supply-side policies [38]. These three policies complement each other and comprehensively stimulate innovation activities.

③Promote enterprises innovation synergistically. These three policies complement and promote each other, optimize the innovation environment, enhance the confidence of enterprises, and enable enterprises to increase input in innovative resources [19]. The innovative resources allow enterprises to apply a single technology to multiple products and fields, achieve application breakthroughs, expand the scope of innovation [50], encourage enterprises to strengthen cooperation in the industrial chain and industry-university-research links, and optimize complementary advantages and win-win cooperation [51]. The three policies work together to guide the willingness and behavior of enterprises innovation, and promote enterprises to integrate and optimize internal and external resources to achieve optimal allocation.

### Mathematical deduction

Considering the long-term profitability of innovation, we use the discounted cash flow method to measure the return of enterprises’ innovation. We use *p* to represent the marginal contribution of the product or service P, while *Q* stands for the sales quantity, and *r* stands for the discount rate (or the capital cost rate). According to the economic theory of negative exponential utility function, and the research of Gupta [52] and Li Enji [53], the return function of technological innovation is expressed as *U*(*π*) = -*e*^-*rπ*^, where *r* is the Arrow-Pratt risk aversion coefficient. Therefore, when there is no government support, the net present value (NPV) of the future cash flow of enterprise technological innovation is:

$$\pi_{0}=E[\int_{t=0}^{\infty }pQe^{-rt}dt]$$ (1)

Government subsidies stimulate enterprises to increase innovation resources and increase innovation input and output [9]. That is to say, government subsidies increase the return of innovation on the original basis (when there is no government support), assuming the increase is *S*. Subsequently, the return of innovation when enterprises benefit from government subsidies is:

$$\pi_{(S}{}_{)}=E[\int_{t=0}^{\infty }pQe^{-rt}dt+S]$$ (2)

Tax incentives reduce the tax burden of innovation and reduce the cost of innovation [21]. As a result, tax incentives increase the marginal contribution per units of products or services by *p*_1_. Therefore, the return of innovation when enterprises benefit from tax incentives can be expressed as:

$$\pi_{\left(T\right)}=E[\int_{t=0}^{\infty }(p+p_{1})\;Qe^{-rt}dt]$$ (3)

Government procurement creates new market demand, exerts a demonstration effect of the purchasing behavior of the entire society, creates a stable market demand environment, and increases the number of innovative sales by *Q*_1_. Therefore, the return of innovation when enterprises benefit from government procurement can be expressed as:

$$\pi_{\left(P\right)}=E[\int_{t=0}^{\infty }p\;(Q+Q_{1})\;e^{-rt}dt]$$ (4)

When enterprises benefit from these three policies simultaneously, policies promote innovation synergistically, significantly enhance the confidence of enterprise, and encourage enterprises to increase innovation input and output. All of these effects allow enterprises to increase the sales volume on the original basis and realize additional return $E\left[\int_{t=0}^{\infty }pQ_{2}e^{-rt}dt\right]$ of product p. Moreover, these three policies work together to induce enterprises to expand their resources and capabilities, strengthen cooperation among enterprises, and motivate enterprises to apply a certain technology to multiple products and multiple fields on the basis of the original R&D products, achieve application breakthroughs, add new products *p*', and realize additional return $E\left[\int_{t=0}^{\infty }p'Q'e^{-rt}dt\right]$. Therefore, the return of innovation supported by the three policies is:

$$\pi_{\left(S,T,P\right)}=E[\int_{t=0}^{\infty }pQe^{-rt}dt+S]+E[\int_{t=0}^{\infty }(p+p_{1})\;Qe^{-rt}dt]+E[\int_{t=0}^{\infty }p\;(Q+Q_{1})\;e^{-rt}dt]+E[\int_{t=0}^{\infty }pQ_{2}e^{-rt}dt]+E[\int_{t=0}^{\infty }p'Q'e^{-rt}dt]$$ (5)

Eq (5) can also be expressed as:

$$\pi_{\left(S,T,P\right)}=\pi_{\left(S\right)}+\pi_{\left(T\right)}+\pi_{\left(P\right)}+\Delta \pi_{\left(S,T,P\right)}$$ (6)

Δ*π*_(*S*,*T*,*P*)_ is the synergy effect of science and technology policies:

$$\Delta \pi_{(S,\;T,\;P}{}_{)}=E[\int_{t=0}^{\infty }pQ_{2}e^{-rt}dt]+E[\int_{t=0}^{\infty }p'Q'e^{-rt}dt]$$ (7)

Therefore, science and technology policies promote and complement each other, and promote the innovation synergistically.

## Methodology

### Sample and data

Zhongguancun Science and Technology Park of China is a national independent innovation demonstration zone. Enterprises in this zone can benefit from a number of policy support measures. Therefore, we select 19,063 firms active in the Zhongguancun Science Park from 2013 to 2018. The data comes from the Beijing Bureau of Statistics. To obtain robust results, the following samples were removed: (1) Enterprises that do not meet accounting standards, such as the values of total assets, revenue, sales income, intangible assets and other indicators are less than zero. (2) Enterprises that are missing data concerning government subsidies, tax incentives and government procurement. (3) Discontinuous enterprises during the sample period. The final sample included 2,592 enterprises and 15,552 observations. In order to eliminate the effect of extreme values, all continuous variables were Winsorized at 1 percent and 99 percent levels.

### Variables

1. Dependent variable. The dependent variable is the innovation performance of enterprises. Referencing Guan and Pang [54], we use the sales revenue of new products to measure innovation performance.
2. Independent variables. Independent variables are science and technology policies, including government subsidies, tax incentives, and government procurement.
  Government subsidies include government funds, special schemes, and other financial policies. Referencing to Chen [1], we measure it by the total amount of government subsidies obtained by enterprises.
  Tax incentives are preferential policies formulated by the government to stimulate innovation, such as income tax, value added tax, business tax, and turnover tax. In relevant studies, tax incentives were mostly measured by preferential tax rate, additional deduction, and other income tax, while ignoring the role of other tax policies. We take different preferential tax policies into account, and measure tax incentives by the total amount of tax relief received by enterprises.
  Government procurement is a policy that allows enterprises to provide new systems, services, and products to the government through a series of processes such as research and development and production. Referring to Aschhoff [33], we measure it by the total amount of government procurement obtained by enterprises.
3. Control variables. Referring to Guerzoni and Raiteri [38], Boeing [9], the control variables are firm size, firm age, leverage, domestic and international industry alliances, profitability, operating income, and the number of employees with undergraduate degrees and above. Specific variables and definitions are shown in Table 1.

**Table 1:** Variables.

Variable type	Variable name	Variable definition
**Dependent variable**	Innovation performance (inper)	ln(total revenue of new product sales+1)
**Independent variable**	Government subsidies (sub)	ln(total amount of government subsidies received by enterprises+1)
	Tax incentives (tax)	ln(total amount of tax relief received by enterprises+1)
	Public procurement (pp)	ln(total amount of government procurement received by enterprises+1)
**Control variable**	Firm size (size)	ln (total assets+1)
	Firm age (age)	ln (time of establishment of a firm+1)
	Leverage (lev)	Total liabilities / total assets
	Industry alliance (group)	Dummy, if a firm join the industry alliance equals 1, otherwise equals 0.
	Profitability (roa)	(total profit + interest income) / total assets
	Revenue (revenue)	ln (revenue+1)
	number of employees with bachelor or above (hedu)	ln (number of employees with bachelor or above+1)

### Models

Referring to Zhu et al. [41] and Wei et al. [48], the synergy effect of science and technology policies is measured according to the interactions of government subsidies, tax incentives, and government procurement. Firstly, we analyze the impact of single policies on innovation. Subsequently, we analyze the synergy effects of two policies. Finally, the synergy effect of government subsidies, tax incentives and government procurement on innovation is analyzed, and the model is constructed as follows:

$$\begin{array}{c} {\mathrm{inper}}_{\mathrm{it}}=\alpha_{1}{\mathrm{sub}}_{\mathrm{it}}+\alpha_{2}{\mathrm{tax}}_{\mathrm{it}}+\alpha_{3}{\mathrm{pp}}_{\mathrm{it}}+\alpha_{4}{\mathrm{sub}}_{\mathrm{it}}\times {\mathrm{tax}}_{\mathrm{it}}+\alpha_{5}{\mathrm{sub}}_{\mathrm{it}}\times {\mathrm{pp}}_{\mathrm{it}}+\alpha_{6}{\mathrm{tax}}_{\mathrm{it}}\times {\mathrm{pp}}_{\mathrm{it}}\\ +\alpha_{7}{\mathrm{sub}}_{\mathrm{it}}\times {\mathrm{tax}}_{\mathrm{it}}\times {\mathrm{pp}}_{\mathrm{it}}+\beta_{i}{\mathrm{controls}}_{\mathrm{it}}+{\mathrm{year}}_{i}+{\mathrm{firm}}_{i}+\varepsilon_{\mathrm{it}} \end{array}$$ (8)

In this equation, *inper* represents innovation performance, *sub*, *tax*, *pp* represent government subsidies, tax incentives, government procurement respectively, *controls* are control variables, *year*_*i*_ and *firm*_*i*_ represent the time effect and firm effect, and *ε* is the random error term.

## Empirical results

### Descriptive statistics and correlation analysis

The descriptive statistics and correlation analysis of the main variables are shown in Table 2. It can be seen that government subsidies, tax incentives, and government procurement policies have different levels. The mean value of government subsidies is 705.27 thousand yuan, indicating that Chinese government subsidies have a certain scale. The mean value of tax incentives is 5723.93 thousand yuan, indicating that tax incentives are more effective in China. The mean value of government procurement is 12.25 thousand yuan, indicating that the degree of government procurement in China needs to be improved. From the correlation test, we can deduce that the correlation coefficients among variables are mostly appropriate, indicating that there is no obvious collinearity.

**Table 2:** Descriptive statistics and correlation analysis.

	Mean	Std. dev.	Min.	Max.	1	2	3	4	5	6	7	8	9	10
**inper**	53450.68	384139.50	0	3594695										
**sub**	705.27	3179.65	0	24945.00	1									
**tax**	5723.93	19006.41	0	140406	0.28	1								
**pp**	12.25	109.19	0	1068.00	0.05	0.10	1							
**age**	16.92	6.88	6.00	47.00	0.13	0.11	0.02	1						
**size**	11.56	2.09	6.59	16.65	0.23	0.39	0.08	0.24	1					
**lev**	0.50	0.42	0.01	2.83	-0.03	-0.07	-0.02	-0.02	-0.13	1				
**group**	0.12	0.33	0	1	0.22	0.17	0.05	0.08	0.18	-0.01	1			
**roa**	0.01	0.20	-1.19	0.46	0.04	0.15	0.01	0.09	0.27	-0.43	0.02	1		
**revenue**	394094.10	1077041	168.00	6700000	0.27	0.56	0.09	0.20	0.51	0.01	0.15	0.11	1	
**hedu**	72.95	220.59	0	1614.00	0.28	0.58	0.12	0.10	0.42	-0.03	0.15	0.05	0.53	1

### Empirical analysis of the synergy effect of science and technology policies

We select the panel data of various enterprises active in Zhongguancun Park from 2013 to 2018. The Hausman test demonstrates that the fixed effect model should be used, and the final empirical results are shown in Table 3.

**Table 3:** Regression results.

	Dependent variable:inper								
	M1	M2	M3	M4	M5	M6	M7	M8	M9
**sub**	0.148*** (10.24)			0.133*** (9.16)	0.068*** (2.76)	0.117*** (7.81)		0.052** (2.10)	0.116*** (7.83)
**tax**		0.125*** (11.27)		0.114*** (10.29)	0.100*** (8.26)		0.113*** (10.08)	0.096*** (7.90)	0.111*** (9.97)
**pp**			0.236*** (7.47)	0.208*** (6.61)		0.106*** (3.02)	0.016 (0.32)	0.008 (0.16)	0.136*** (3.96)
sub*tax					0.049*** (3.47)			0.040*** (2.80)	
sub*pp						0.326*** (7.21)		0.280*** (5.85)	
tax*pp							0.169*** (5.30)	0.083** (2.44)	
sub*tax*pp									0.161*** (5.25)
**controls**	control	control	control	control	control	control	control	control	control
**firm effect**	yes	yes	yes	yes	yes	yes	yes	yes	yes
**time effect**	yes	yes	yes	yes	yes	yes	yes	yes	yes
**c**	-7.568*** (-21.66)	-7.379*** (-21.05)	-7.796*** (-22.35)	-7.095*** (-20.26)	-7.090*** (-20.22)	-7.492*** (-21.51)	-7.280*** (-20.80)	-7.020*** (-20.08)	-7.075*** (-20.23)
**observations**	15552	15552	15552	15552	15552	15552	15552	15552	15552
**R2**	0.10	0.10	0.10	0.11	0.11	0.11	0.11	0.12	0.11
**F**	228.50	213.58	221.70	200.15	196.58	194.11	193.89	159.75	184.77

Note

“***”, “**” and “*”mean significant at the level of 1%, 5% and 10% respectively.

It is apparent that the coefficients of government subsidies, tax incentives, and government procurement on innovation performance are all significantly positive (the coefficients are 0.148, 0.125, and 0.236, respectively). That is, single policies all demonstrate positive incentive effects on innovation.

Of the two policies, the coefficient of the interaction of government subsidies and government procurement is significantly positive (0.326), indicating that government subsidies and government procurement exert a synergy effect in the process of stimulating enterprise innovation. The two policies compliment and promote each other, and stimulate innovation synergistically. The coefficient of the interaction between tax incentives and government procurement is also significantly positive (0.169), indicating that tax incentives and government procurement also exert synergy effect during the process of stimulating enterprises’ innovation. The coefficient of the interaction of government subsidies and tax incentives is significantly positive, indicating that government subsidies and tax incentives do exert a certain synergy effect during the process of innovation.

Subsequently, we further analyze the synergy effect of three policies. The result demonstrate that the coefficient of the intersection is significantly positive (0.161), indicating that one policy is influenced by the other two policies, and that the three policies interact each other to exert synergy effect in promoting innovation.

### Robustness test

1. Innovation performance is measured by the sales revenue of new products, and 68 percent of the observations in the samples are zero. In order to overcome the problem of biased estimation caused by the merging of data, the random effect tobit model was applied. The results are generally consistent with the benchmark results (S1 Appendix).
2. In this paper, the number of patent applications is used as an alternative variable of innovation performance for the robustness test. The results are generally consistent with the benchmark results, too (S2 Appendix).
3. In September 2014, Premier Li Keqiang proposed “Mass Entrepreneurship” and “Millions of Innovations”, and formally proposed “Mass Entrepreneurship and Innovations” in the “Government Work Report” in 2015, In order to eliminate the influence of the change of the policy implementation background on the results, we take 2015 as a watershed for grouping test and find that the regression results before and after 2015 are all consistent with the benchmark results, which indicated that the results are robust (S3 Appendix).

### The realization path of the synergy effect

Innovation is a dynamic process with multiple stages, then, what is the realization path of the synergy effect of science and technology policies in the process of innovation? In other words, how do government subsidies, tax incentives, and government procurement interact and function during different stages of innovation?

The innovation cycle includes the creation of ideas, research and development, the intermediate output of patents, and the final output of new products on the market. In the relevant research, Guan [54] measured the innovation by innovation tendency, technological performance, and economic performance. Li [55] divided the innovation process into technological development and achievement transformation. According to the innovation cycle and the existing research, we divide innovation into three processes: innovation input, technology development, and achievement transformation. Innovation input is the input stage during which enterprises participate in innovation. This process reflects the degree of enterprises' tendency and willingness to participate in innovation, and is measured by the amount of enterprise’s R&D expenditures. Technology development is the stage of carrying out of new knowledge, new process and new technology, during this stage, enterprises can realize technological achievements through innovation, and is measured by the number of patent applications submitted by enterprises. During the achievement transformation stage, new knowledge, new processes and new technology are applied to the development of new products and the realization of commercial applications. This stage represents the final achievement transformation of innovation, and is measured by the sales revenue of new products.

**Fig 1:**
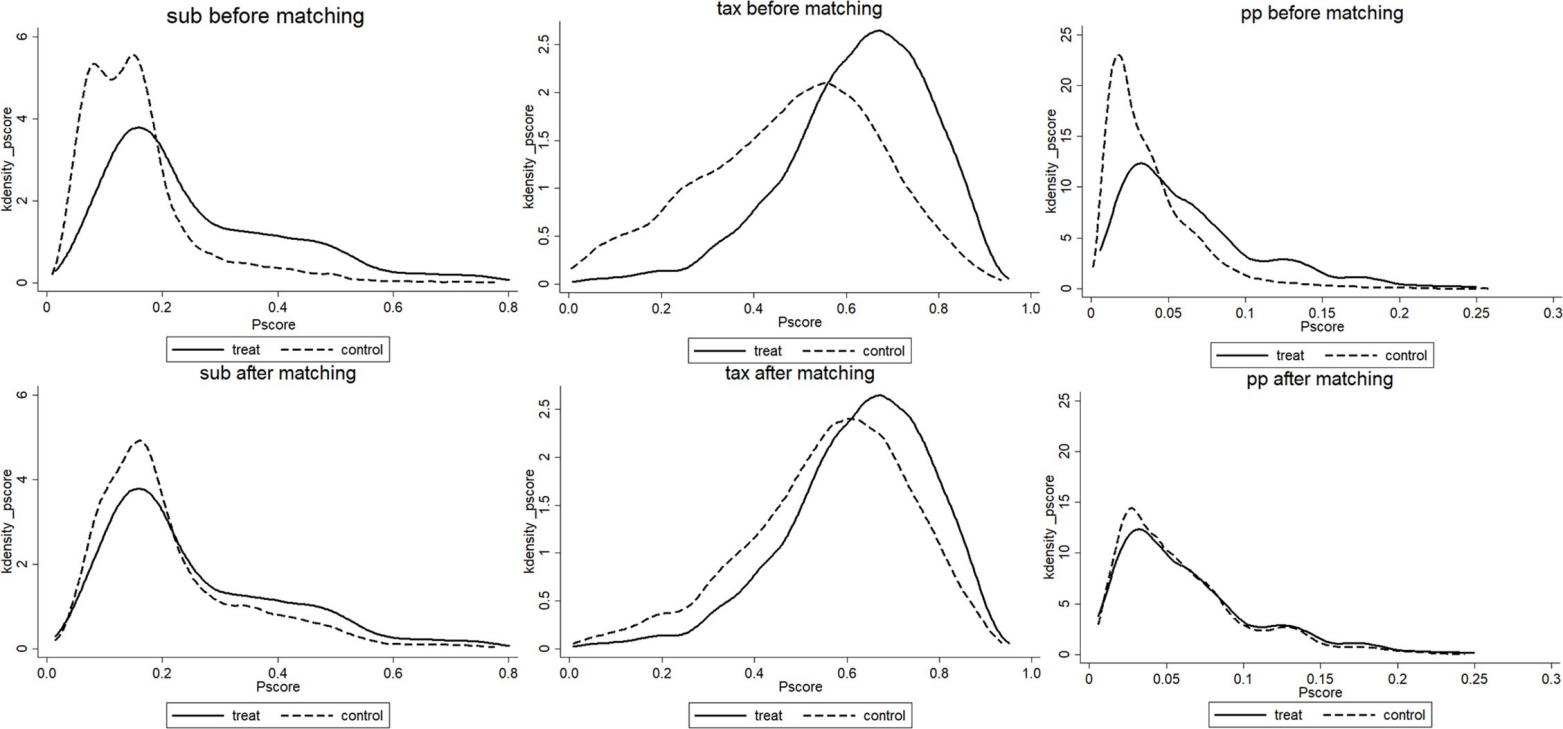
Density function graph before and after Match.

Considering the sample selection problem, we analyze the path of synergy effect by the propensity score matching method. Independent variables consist of government subsidies, tax incentives and government procurement. dependent variables consist of R&D expenditure, the number of patent applications, and sales revenue of new products. Control variables are firm age, firm size and others. The variables and data are identical to those listed above. The matching balance test proves that the matching result is appropriate (Fig 1). The results of the propensity score are shown in Table 4.

**Table 4:** PSM results of the realization path of science and technology policies.

	Dependent variable					
	R&D expenditure	number of patent applications	sales revenue of new products	Lag 1 period		
				R&D expenditure	number of patent applications	sales revenue of new products
sub	0.524*** (5.00)	0.576*** (15.67)	1.217*** (7.55)	0.863*** (8.96)	0.546*** (12.88)	0.918*** (4.77)
tax	0.196*** (2.67)	0.167*** (6.88)	1.057*** (11.40)	0.139 (1.64)	0.096*** (3.57)	0.849*** (8.47)
pp	0.202 (1.33)	0.149** (2.08)	2.246*** (6.52)	0.177 (0.93)	0.178** (2.36)	1.418*** (4.21)
controls	control	control	control	control	control	control
firm effect	control	control	control	control	control	control
time effect	control	control	control	control	control	control

Note

“***”, “**” and “*”mean significant at the level of 1%, 5% and 10% respectively.

The results demonstrate that the policies have stable incentive effects on innovation input, technology development, and achievement transformation. The coefficients of government subsidies, tax incentives, and government procurement on R&D expenditure are 0.524, 0.196, and 0.202, respectively. The coefficient of the government subsidies is the largest, therefore, government subsidies are shown to have the strongest incentive effects on R&D expenditure. Similarly, government subsidies have the strongest incentive effects on the number of patent applications and government procurement has the strongest incentive effect on the sales revenue of new products. Stated differently, government subsidies have the strongest incentive effects on innovation input and technology development; government procurement have the strongest incentive effect on the transformation stage; and tax incentives have a balanced effect on innovation during all stages. Considering that it takes considerable time for technology to be materialize from research and development, the outcome variables are processed with a lag of one period. These robust results are displayed in the last three columns of Table 6, and the results are robust.

Therefore, during the process of innovation, government subsidies exert the strongest effects in the stages of innovation input and technology development, and.government procurement exert the strongest effect on the transformation stage. Therefore, with regards to innovation, policies should be utilized for their unique advantages and functions specific to different stages of innovation. This way, they can strengthen incentive effects during the entire process of innovation, and stimulate innovation synergistically.

## Mechanisms of the synergy effect of science and technology policies

According to theoretical analysis, policies stimulate innovation by increasing investment in innovation resources, expanding innovation scope, and increasing innovation cooperation. Simply put, innovation resource input, innovation technology spillover, and innovation cooperation play mediating roles in science and technology policies and innovation performance, which will be verified below.

### The mediating role of innovative resource input

When government subsidies, tax incentives, and government procurement policies support enterprises simultaneously, it will send a strong signal of government support to the public, which attracts investment [31], and alleviates corporate financing constraints [32]. At the same time, the new products will be more widely recognized in the market because of government support, which stimulates other market players to buy new products and leads to a larger demand for products [31]. The three policies work together to encourage enterprises to increase innovation resource input in an comprehensive manner [2]. The scale input of innovation resources will certainly provide a more adequate guarantee for enterprises to improve product functions and technical content, promote enterprises to integrate and optimize various resources, increases output, and improves performance [38]. Therefore, the input of innovation resources plays a mediating role between science and technology policies and innovation performance.

Innovation resource inputs includes capital, talent, technology, policy, knowledge, etc. We take capital as an indicator of innovation resource input and measure it by the capital expenditure of innovation (finan). Referring to Tang and Wu [56], science and technology policies (sub_tax_pp) are measured with a dummy variable. If enterprises receive government subsidies, tax incentives and policy procurement at the same time, the value is 1; otherwise, it is 0. We subsequently select science and technology policies as the independent variable, innovation performance as the dependent variable; and firm size, age, leverage, profitability, and industry alliance as control variables to examine the mediating effect of innovation resource input by the three steps intermediary regression analysis method. The sample and data are the same as above. The models are as follows:

$$\begin{array}{l} \mathrm{inper}=\alpha_{1}\mathrm{sub}\_\mathrm{tax}\_\mathrm{pp}+\beta_{i}\mathrm{controls}+{\mathrm{year}}_{i}+firm_{i}+\varepsilon \\ \mathrm{finan}=\alpha_{2}\mathrm{sub}\_\mathrm{tax}\_\mathrm{pp}+\beta_{i}\mathrm{controls}+{\mathrm{year}}_{i}+firm_{i}+\varepsilon \\ \mathrm{inper}=\alpha_{3}\mathrm{sub}\_\mathrm{tax}\_\mathrm{pp}+\alpha_{4}\mathrm{finan}+\beta_{i}\mathrm{controls}+{\mathrm{year}}_{i}+firm_{i}+\varepsilon \end{array}$$ (9)

The regression results of multi-level panel fixed effect are shown in Table 5.

**Table 5:** Mediating effect test results of innovation resource input.

	inper M1	finan M2	inper M3
**sub_tax_pp**	3.593*** (10.04)	0.777*** (6.50)	3.561*** (9.94)
**finan**			0.040* (1.68)
**controls**	control	control	control
**firm effect**	yes	yes	yes
**time effect**	yes	yes	yes
**c**	-7.728*** (-22.18)	1.479*** (12.71)	-7.788*** (-22.23)
**R2**	0.10	0.04	0.10
**F**	227.92	1407.61	202.93

Note

“***”, “**” and “*”mean significant at the level of 1%, 5% and 10% respectively.

The results show that science and technology policies have a significant positive effect on innovation performance (α1 = 3.593, p<0.001). Further we examine the impact of science and technology policies on innovation resource input, the result show that science and technology policies is significantly positively correlated with innovation resource input (α2 = 0.777, p<0.001). Subsequently, we test the mediating role of innovation resource input, when science and technology policies and innovation resource input are are put in the regression of innovation performance simultaneously, innovation resource input has a considerably positive effect on innovation performance (α4 = 0.040, p<0.1). Science and technology policies also demonstrate a considerably positive effect (α3 = 3.561, p<0.001). However, α3<α1 which shows that innovation resource input plays a partial mediating role in science and technology policies and innovation performance, namely the synergy effect of the science and technology policies on innovation is realized through the scale effect of innovation resource input.

### The mediating role of innovation technology spillover

Innovative technology is derivative. Technological progress will promote the integration and optimization of funds, talents, technology and other resources. Science and technology policies are conducive to the optimization of internal resources of enterprises, and allow enterprises to take advantage of external advantaged resources, motivate enterprises to apply innovative technology to related products, and achieve application breakthroughs, which makes the scope of innovation extends from the inherent innovation field to new types of products. The spillover of innovation technology expands the scope of enterprise innovation, enables enterprises to realize resource and cost sharing among products linked by research and development, production, operation and sales, so as to achieve better innovation output and improve innovation performance [57]. Therefore, the spillover of innovative technology mediates the science and technology policies and innovation performance.

Innovative technology spillover is the expansion of the scope of enterprise technology innovation, and is measured by the number of new technology fields of patent application in a year. Referring to Yu [58], we use the first four digits of the main classification number of international patent classification (IPC) to represent a certain technical field. Patent data is collected from the official website of the state intellectual property office. Other data and variables are the same as above. We subsequently examine the mediating effect of innovative technology spillover. The test process is the same as described above, and the multi-level panel fixed effect regression results are shown in Table 6.

**Table 6:** Mediating effect test results of innovative technology spillover.

	inper M1	spillover M2	inper M3
**sub_tax_pp**	3.593*** (10.04)	0.706*** (6.83)	3.295*** (9.26)
**spillover**			0.422*** (15.28)
**controls**	control	control	control
**firm effect**	yes	yes	yes
**time effect**	yes	yes	yes
**c**	-7.728*** (-22.18)	-1.888*** (-18.78)	-6.932*** (-19.82)
**R2**	0.10	0.14	0.12
**F**	227.92	386.34	231.56

Note

“***”, “**” and “*”mean significant at the level of 1%, 5% and 10% respectively.

We can find that science and technology policies are significantly positively correlated with innovation performance (α1 = 3.593, p<0.001). Furthermore, we explore the relationship between science and technology policies and innovation technology spillover. The results show that science and technology policies exert a significant positive effect on innovation technology spillover (α2 = 0.706, p<0.001). We also test the mediating role of innovation technology spillover and determine that when science and technology policies and innovation technology spillover are put in the regression of innovation performance simultaneously, innovation technology spillover has a significant positive effect on innovation performance (α4 = 0.422, p< 0.001). Science and technology policies also has a significant positive effect (α3 = 3.295, p<0.001), however, α3<α1, demonstrating that innovation technology spillover plays a partial mediating role between science and technology policies and innovation performance.

### The mediating role of innovation cooperation

Science and technology policies compliment and promote each other, and synergistically motivate enterprises to expand resources and capabilities. But due to the constraints of internal resources, technology, knowledge and other internal resources, enterprises start to seek cooperation with others with different backgrounds, different talents, different links in the field of technology to strengthen the cooperation along the industrial chain and the industry-university-research chain, and realize technology innovation in the form of "union", namely science and technology policies promote enterprise to strengthen innovation cooperation [51]. Innovation cooperation enables enterprises to benefit from more heterogeneous resources and obtain "relational rent" from shared resources, thus realize complementary advantages and win-win cooperation, all of which improve innovation performance [59]. Therefore, innovation cooperation mediates the synergy effect of science and technology policies and innovation performance.

Referring to Li [60], innovation cooperation (incoopera) is measured by the amount of innovation cooperation expenditure of enterprises. Other variables and samples are the same as above. Results are shown in Table 7.

**Table 7:** Mediating effect test results of innovation cooperation.

	inper M1	incoopera M2	inper M3
sub_tax_pp	3.593*** (10.04)	2.024*** (9.62)	3.427*** (9.56)
incoopera			0.082*** (6.01)
controls	control	control	control
firm effect	yes	control	control
time effect	yes	control	control
c	-7.728*** (-22.18)	-1.603*** (-7.83)	-7.596*** (-21.78)
R2	0.10	0.05	0.10
F	227.92	104.45	207.07

Note

“***”, “**” and “*”mean significant at the level of 1%, 5% and 10% respectively.

After controlling for firm age, firm size, firm leverage, industrial alliances, profitability, employees with a bachelor’s degree or above, science and technology policies are shown to have a significant positive impact on innovation performance (α1 = 3.593, p<0.001). Furthermore, we find that science and technology policies positively affect innovation cooperation (α2 = 2.024, p<0.001). Moreover, when science and technology policies and innovation cooperation are simultaneously placed in the regression of innovation performance, innovation cooperation has a significant positive effect on innovation performance (α4 = 0.082, p<0.001), science and technology policies also have a significant positive effect (α3 = 3.427, p<0.001), however α3<α1, demonstrating that innovation cooperation plays a partial mediating role in science and technology policies and innovation performance.

## Conclusions and suggestions

### Conclusions

Under the background of the strategy of innovation-driven development, government makes a series of science and technology policies to support innovation. Science and technology policies interact with each other and synergistically promote enterprise innovation. We take government subsidies, tax incentives and government procurement as the research object, and attempt an empirical analysis of the synergy effect of science and technology policies on innovation, and further analyze the path and mechanisms of the synergy effect. The conclusions are as follows:

1. Government subsidies, tax incentives and government procurement all exert incentive effects on innovation. The three policies complement and promote each other, synergistically stimulate innovation.
2. In the process of the technology innovation, the effects of government subsidies on the stage of innovation input and technology development are the strongest, the effect of government procurement on the stage of achievements transformation is the strongest, and the tax incentives is balanced and stable in the whole process of technology innovation, the three policies are corresponding, orderly and continuous in the process of innovation.
3. Innovation resource input, innovation technology spillover, and innovation cooperation all play partial mediating roles between science and technology policies and innovation performance. Stated differently, the synergy effect of science and technology policies encourage innovation by increasing innovation resource input, realizing spillover of innovation technologies, and enhancing innovation cooperation.

### Suggestions

Based on the above results, it is necessary to formulate targeted and multi-level policies responsive to the characteristics of innovation in different stages.

1. Government should improve the structural system of science and technology policies, optimize synergy effect, and oversee an effective combination of science and technology policies. Technological innovation policies should be diversified and optimized according to the characteristics of different stages of innovation, the level of synergy effect, and the status of technology in the industry, and formulate diversified policy system of the supply side and the demand side. At the same time, governments should implement environmental construction policies such as intellectual property protection, remove institutional barriers to independent innovation, and improve the performance of policies and the level of the synergy effect.
2. Government should plan policies to achieve precise support for innovation. Different policies should be utilized to stimulate enterprise innovation responsive to the different stages of innovation. Government subsidies should be provided to enterprises which are in the stages of innovation input and technology development. Government procurement should be offered to enterprises that are in the stage of achievement transformation. At the same time, tax incentives should be further improved to strengthen the convergence, complementation and the linkage of policies, so as to give full play to the best implementation of science and technology policies.
3. Government must strengthen policies to promote innovation cooperation along the industrial and industry-university-research chain, and promote technology integration and cross-border development. Pursuing the goal of cross-border integration breakthrough, government should build innovation cooperation platforms for essential cutting-edge technologies, formulate a joint innovation and sharing mechanism of “industry-university-research institute”, speed up the integration of innovation technology and diffusion of different areas, different industries and different links, and form new technology innovation "unions" to stimulate technology convergence and crossover development.

## Supporting information

S1 Appendix Tobit regression results. (DOC)

S2 Appendix Regression results (number of patent applications). (DOC)

S3 Appendix Grouped regression results. (DOC)

S1 Data (XLS)
