# Supplementary material for: Synergy effect of science and technology policies on innovation: Evidence from China
Source: PLoS One. 2020 Oct 13;15(10):e0240515. doi: 10.1371/journal.pone.0240515 (PMC7553322; doi:10.1371/journal.pone.0240515)
Supplement: S1 Appendix — (DOC) [file pone.0240515.s001.doc]

**S1 Appendix. Tobit regression results**

|  | **Dependent variable:inper** | | | | | | | |
| --- | --- | --- | --- | --- | --- | --- | --- | --- |
|  | **M1** | **M2** | **M3** | **M4** | **M5** | **M6** | **M7** | **M8** |
| **sub** | 0.307***  （7.44） |  |  | 0.264***  （6.41） | 0.225***  （3.05） | 0.243***  （5.69） |  | 0.240***  （5.67） |
| **tax** |  | 0.333***  （10.01） |  | 0.308***  （9.26） | 0.302***  （8.25） |  | 0.310***  （9.20） | 0.303***  （9.08） |
| **pp** |  |  | 0.432***  （4.98） | 0.367***  （4.26） |  | 0.183*  （1.85） | 0.066  （0.48） | 0.266***  （2.80） |
| **sub*tax** |  |  |  |  | 0.032  （0.78） |  |  |  |
| **sub*pp** |  |  |  |  |  | 0.570***  （4.70） |  |  |
| **tax*pp** |  |  |  |  |  |  | 0.267***  （3.08） |  |
| **sub*tax*pp** |  |  |  |  |  |  |  | 0.230**  （2.57） |
| **controls** | control | control | control | control | control | control | control | control |
| **firm effect** | yes | yes | yes | yes | yes | yes | yes | yes |
| **time effect** | yes | yes | yes | yes | yes | yes | yes | yes |
| **c** | -32.75***  (-29.13) | -32.15***  (-28.57) | -33.37***  (-29.68) | -31.26***  (-27.85) | -31.41***  (-27.94) | -32.48***  (-28.98) | -31.83***  (-28.35) | -31.23***  (-27.84) |
| **Pseudo R2** | 0.03 | 0.03 | 0.03 | 0.03 | 0.03 | 0.03 | 0.03 | 0.25 |
| **LR chi2（8）** | 1268.64 | 1314.53 | 1237.98 | 1375.94 | 1358.71 | 1311.49 | 1344.53 | 1382.49 |
| **Prob>chi2** | 0 | 0 | 0 | 0 | 0 | 0 | 0 | 0 |

Note: “***”, “**” and “*”mean significant at the level of 1%, 5% and 10% respectively.
