# Supplementary material for: Synergy effect of science and technology policies on innovation: Evidence from China
Source: PLoS One. 2020 Oct 13;15(10):e0240515. doi: 10.1371/journal.pone.0240515 (PMC7553322; doi:10.1371/journal.pone.0240515)
Supplement: S2 Appendix — (DOC) [file pone.0240515.s002.doc]

**S2 Appendix. regression results ( number of patent applications )**

|  | **Dependent variable: patent** | | | | | | | |
| --- | --- | --- | --- | --- | --- | --- | --- | --- |
|  | **M1** | **M2** | **M3** | **M4** | **M5** | **M6** | **M7** | **M8** |
| **sub** | 0.091***  （27.28） |  |  | 0.088***  （26.45） | 0.072***  （12.86） | 0.088***  （25.59） |  | 0.087***  （25.47） |
| **tax** |  | 0.028***  （10.78） |  | 0.022***  （8.85） | 0.019***  （6.81） |  | 0.027***  （10.18） | 0.022***  （8.73） |
| **pp** |  |  | 0.032***  （4.38） | 0.020***  （2.83） |  | 0.013*  （1.65） | 0.010  （0.87） | 0.015*  （1.91） |
| **sub*tax** |  |  |  |  | 0.011***  （3.47） |  |  |  |
| **sub*pp** |  |  |  |  |  | 0.027***  （2.64） |  |  |
| **tax*pp** |  |  |  |  |  |  | 0.015***  （2.07） |  |
| **sub*tax*pp** |  |  |  |  |  |  |  | 0.012***  （1.71） |
| **controls** | control | control | control | control | control | control | control | control |
| **firm effect** | yes | yes | yes | yes | yes | yes | yes | yes |
| **time effect** | yes | yes | yes | yes | yes | yes | yes | yes |
| **c** | -1.848***  (-23.15) | -1.920***  (-23.50) | -2.020***  (-24.81) | -1.760***  (-21.96) | -1.753***  (-21.87) | -1.840***  (-23.06) | -1.909***  (-23.36) | -1.758***  (-21.94) |
| **R2** | 0.24 | 0.21 | 0.20 | 0.25 | 0.25 | 0.24 | 0.21 | 0.25 |
| **F** | 675.75 | 574.74 | 559.15 | 552.46 | 553.00 | 542.84 | 462.29 | 502.56 |

Note: “***”, “**” and “*”mean significant at the level of 1%, 5% and 10% respectively.
