# Supplementary material for: Synergy effect of science and technology policies on innovation: Evidence from China
Source: PLoS One. 2020 Oct 13;15(10):e0240515. doi: 10.1371/journal.pone.0240515 (PMC7553322; doi:10.1371/journal.pone.0240515)
Supplement: S3 Appendix — (DOC) [file pone.0240515.s003.doc]

**S3 Appendix. Grouped regression results**

| **Panel1: Regression results before 2015** | | | | | | | |
| --- | --- | --- | --- | --- | --- | --- | --- |
|  | **M1** | **M2** | **M3** | **M4** | **M5** | **M6** | **M7** |
| **sub** | 0.095***  （5.32） |  |  | 0.030  （1.02） | 0.055***  （2.96） |  | 0.058***  （3.19） |
| **tax** |  | 0.091***  （6.34） |  | 0.071***  （4.53） |  | 0.087***  （5.99） | 0.086***  （6.05） |
| **pp** |  |  | 1.022***  （14.73） |  | 0.811***  （9.97） | 0.859***  （8.85） | 0.910***  （12.01） |
| **sub*tax** |  |  |  | 0.044**  （2.48） |  |  |  |
| **sub*pp** |  |  |  |  | 0.244***  （4.50） |  |  |
| **tax*pp** |  |  |  |  |  | 0.098**  （2.49） |  |
| **sub*tax*pp** |  |  |  |  |  |  | 0.118***  （3.24） |
| **c** | -6.715***  (-15.97) | -6.551***  (-15.51) | -6.406***  (-15.41) | -6.350***  (-15.00) | -6.282***  (-15.11) | -6.023***  (-14.41) | -5.930***  (-14.18) |
| **controls** | control | control | control | control | control | control | control |
| **firm effect** | yes | yes | yes | yes | yes | yes | yes |
| **time effect** | yes | yes | yes | yes | yes | yes | yes |
| **Observations** | 7776 | 7776 | 7776 | 7776 | 7776 | 7776 | 7776 |
| **R2** | 0.12 | 0.12 | 0.14 | 0.13 | 0.15 | 0.15 | 0.15 |
| **Panel2: Regression results after 2015** | | | | | | | |
| **sub** | 0.222***  （9.64） |  |  | 0.133***  （3.32） | 0.208***  （8.82） |  | 0.193***  （8.21） |
| **tax** |  | 0.171***  （10.23） |  | 0.143***  （7.84） |  | 0.167***  （9.85） | 0.156***  （9.32） |
| **pp** |  |  | 0.081**  （2.15） |  | 0.017  （0.39） | -0.042（-0.59） | 0.005  （0.11） |
| **sub*tax** |  |  |  | 0.047**  （2.14） |  |  |  |
| **sub*pp** |  |  |  |  | 0.182**  （2.28） |  |  |
| **tax*pp** |  |  |  |  |  | 0.086  （1.61） |  |
| **sub*tax*pp** |  |  |  |  |  |  | 0.100*  （1.97） |
| **c** | -8.335***  (-14.73) | -8.133***  (-14.34) | -8.809***  (-15.54) | -7.717***  (-13.64) | -8.347***  (-14.76) | -8.145***  (-14.37) | -7.765***  (-13.72) |
| **controls** | control | control | control | control | control | control | control |
| **firm effect** | yes | yes | yes | yes | yes | yes | yes |
| **time effect** | yes | yes | yes | yes | yes | yes | yes |
| **Observations** | 7776 | 7776 | 7776 | 7776 | 7776 | 7776 | 7776 |
| **R2** | 0.11 | 0.11 | 0.10 | 0.12 | 0.11 | 0.11 | 0.12 |

Note: “***”, “**” and “*”mean significant at the level of 1%, 5% and 10% respectively.
